# Supplementary material for: Dietary Factors of blaNDM Carriage in Health Community Population: A Cross-Sectional Study
Source: Int J Environ Res Public Health. 2021 Jun 2;18(11):5959. doi: 10.3390/ijerph18115959 (PMC8199633; doi:10.3390/ijerph18115959)
Supplement: Supplementary file 1 [file ijerph-18-05959-s001.zip › ijerph-1196462-supplementary.pdf]

Table S1 Baseline demographics of the participants with or without fecal carriage of *bla*$_{NDM}$ gene.

Variables	Rank	All participants (*n* = 515)	*bla*$_{NDM}$ carrier (*n* = 99)	*bla*$_{NDM}$ non-carrier (*n* = 416)	*P*-value
Stature (cm)		159.04±10.68	160.79±9.02	158.61±11.00	0.068
Weight (kg)		61.14±10.49	63.09±10.71	60.67±10.39	0.039*
Waist-hip ratio		0.89±0.08	0.90±0.08	0.89±0.08	0.303
Muscle mass (kg)		41.25±8.46	42.93±9.18	40.85±8.24	0.028*
Basal metabolic rate (BMR) (kcal)		1254.70±229.72	1300.72±254.13	1243.58±222.34	0.026*
Transfusion, n(%)	No	512(99.4)	98(99.0)	414 (99.5)	0.534
	Yes	3(0.6)	1(1.0)	2(0.5)	
Intravenous drip, n(%)	No	488(94.8)	94(94.9)	394(94.7)	0.924
	Yes	27(5.2)	5(5.1)	22(5.3)	
Oxygen mask, n(%)	No	511(99.2)	98(99.0)	413(99.3)	0.768
	Yes	4(0.8)	1(1.0)	3(0.7)	
Cardiac Surgery, n(%)	No	515(100.0)	99(100.0)	416(100.0)	-
	Yes	0(0.0)	0(0.0)	0(0.0)	
Oral medicine in recent three months, n(%)	No	185(35.9)	37(37.4)	148(35.6)	0.769
	Yes	327(63.5)	61(61.6)	266(81.3)	
	Not clear	3(0.6)	1(1.0)	2(0.5)	
Surgery, n(%)	No	507(98.4)	96(97.0)	411(98.9)	0.406
	Yes	5(1.0)	2(2.0)	3(60.0)	
	Not clear	3(0.6)	1(1.0)	2(0.5)	
Hospitalization, n(%)	No	504(97.9)	96(97.0)	408(98.1)	0.753
	Yes	8(1.6)	2(2.0)	6(1.4)	
	Not clear	3(0.6)	1(1.0)	2(0.5)	
Visiting patients in recent three months, n(%)	No	422(81.9)	79(79.8)	343(82.5)	0.537
	Yes	93(18.1)	20(20.2)	73(17.5)	
Source of drinking water, n(%)	Tap-water	470(91.3)	88(88.9)	382(91.8)	0.352
	Commercial bottled water	45(8.7)	11(11.1)	34(8.2)	
Water filter (if drinking tap-water), n(%)	No	215(41.8)	40(40.4)	175(42.2)	0.749
	Yes	299(58.2)	59(59.6)	240(57.8)	
Sleep quality, n(%)	Good	155(30.2)	36(36.7)	119(28.7)	0.186

	General	269(52.4)	49(50.0)	220(53.0)	
	Not good	69(13.5)	8(8.2)	61(14.7)	
	Chronic Insomnia	20(3.9)	5(5.1)	15(3.6)	
Appetite for salty food, n(%)	Not salty	199(38.6)	34(34.3)	165(39.7)	0.054
	General	213(41.4)	38(38.4)	175(42.1)	
	Salty	102(19.8)	26(26.3)	76(18.3)	
	Extensive salty	1(0.2)	1(1.0)	0(0.0)	
Appetite for spicy food, n(%)	Not spicy	279(54.3)	53(53.5)	226(54.5)	0.962
	General	179(34.8)	34(34.3)	145(34.9)	
	Spicy	50(9.7)	11(11.1)	39(9.4)	
	Extensive spicy	6(1.2)	1(1.0)	5(1.2)	
Appetite for sweet food, n(%)	Not sweet	338(66.1)	66(66.7)	272(66.0)	0.307
	General	126(24.7)	20(20.2)	106(25.7)	
	Sweet	42(8.2)	11(11.1)	31(7.5)	
	Extensive sweet	5(1.0)	2(2.0)	3(0.7)	
Cholesterol	< 2.9 mmol/L	1(0.2)	0(0.0)	1(0.2)	0.351
	2.9 ~ 5.2 mmol/L	371(72.0)	66(66.7)	305(73.3)	
	> 5.2 mmol/L	143(27.8)	33(33.3)	110(26.4)	
High density lipoprotein	<1.16mmol/L(male) <1.29 mmol/L(female)	0(0.0)	0(0.0)	0(0.0)	0.534
	1.16~1.42 mmol/L(male) 1.29~1.55 m mol/L(female)	83(16.1)	18(18.2)	65(15.6)	
	>1.42 mmol/L (male) >1.55 m mol/L (female)	432(83.9)	81(81.8)	351(84.4)	
Low density lipoprotein	< 2.6 mmol/L	286(55.5)	51(51.5)	235(56.5)	0.596
	2.6~ 3.4mmol/L	145(28.2)	29(29.3)	116(27.9)	
	>3.4 mmol/L	84(16.3)	19(3.7)	65(12.6)	

Fasting insulin	< 2.6 μU/mL	7(1.4)	1(1.0)	6(1.4)	0.329
	2.6~ 24.9 μU/mL	497(96.5)	94(94.9)	403(96.9)	
	>24.9 μU/mL	11(2.1)	4(36.4)	7(1.7)	
Insulin resistance index	< 4.0%	0(0.0)	0(0.0)	0(0.0)	-
	4.0~ 6.0%	515(100.0)	99(100.0)	416(100.0)	
	>6.0%	0(0.0)	0(0.0)	0(0.0)	
Antibiotic usage in recent three months, n(%)					
Penicillin	No	482(94.5)	98(100.0)	384(93.2)	0.008**
	Yes	28(5.5)	0(0.0)	28(6.8)	
Floxacin	No	504(98.8)	95(96.9)	409(99.3)	0.054
	Yes	6(1.2)	3(3.1)	3(0.7)	
Polymyxin	No	509(99.8)	97(99.0)	412(100.0)	0.040*
	Yes	1(0.2)	1(1.0)	0(0.0)	
Metronidazole	No	500(98.0)	98 (100.0)	402(97.6)	0.119
	Yes	10(2.0)	0(0.0)	10(2.4)	
Cephalosporins	No	471(92.4)	90(91.8)	381(92.5)	0.831
	Yes	39(7.6)	8(8.2)	31(7.5)	
Streptomycin	No	508(99.6)	98(100.0)	410(99.5)	0.490
	Yes	2(0.4)	0(0.0)	2(0.5)	
Tetracycline	No	510(100.0)	98(100.0)	412(100.0)	-
	Yes	0(0.0)	0(0.0)	0(0.0)	
Erythromycin	No	498(97.6)	96(98.0)	402(97.6)	0.821
	Yes	12(2.4)	2(2.0)	10(2.4)	
Carbopenems	No	510(100.0)	98(100.0)	412(100.0)	-
	Yes	0(0.0)	0(0.0)	0(0.0)	
Glycopeptides	No	510(100.0)	98(100.0)	412(100.0)	-
	Yes	0(0.0)	0(0.0)	0(0.0)	

ANOVA was used for statistical comparisons between quantitative variables, and chi-square test was used for statistical comparisons between qualitative variables.

Weighted values are means and their SD (continuous variables) or percentages (categorical variables).

*p<0.05, **p<0.01 significantly associated with prevalence of bla_{NDM} carrier.

-, No statistics were calculated for the statistic is constant.

Table S2 Spearman's rank correlation between the different food groups

	Vegetable oil	Animal fats	Pork	Beef	Mutton	Poultry	Gamey meat	Animal innards	Freshwater fish	Marine fish	Egg	Leafy vegetables	Root & tuber crops	Fruit	Rice	Wheat	Coarse grain	Milk or milk-products	Yogurt	Soybean and its product
Vegetable oil	1.000	-0.021	0.118**	-0.007	0.051	0.033	0.017	0.105*	-0.001	0.066	0.063	0.348**	0.014	0.085	0.265**	0.144**	0.056	-0.089*	-0.053	-0.056
Animal fats		1.000	0.193**	0.202**	0.149**	0.107*	0.177**	0.306**	0.167**	0.170**	0.033	-0.011	0.138**	0.025	0.127**	-0.022	0.028	0.075	0.094*	0.063
Pork			1.000	0.209**	0.150**	.330**	0.055	0.210**	0.228**	0.208**	0.110*	0.083	0.117**	-0.007	0.320**	0.063	0.021	0.097*	-0.024	0.046
Beef				1.000	.402**	0.260**	0.140**	0.174**	0.260**	0.307**	0.037	0.065	0.111*	0.104*	0.078	-0.001	0.102*	0.086	0.117**	0.074
Mutton					1.000	0.237**	0.134**	0.157**	0.101*	0.217**	0.062	0.054	0.081	0.083	-0.007	0.034	0.127**	0.153**	0.192**	0.040
Poultry						1.000	0.081	0.244**	0.288**	0.303**	.137**	0.155**	0.077	0.065	0.097*	0.082	0.109*	0.102*	0.070	0.055
Gamey meat							1.000	0.245**	0.088*	0.144**	-0.007	-0.054	0.029	0.034	0.061	-0.078	-0.067	0.053	0.084	0.053
Animal innards								1.000	0.054	0.292**	0.023	0.044	0.090*	0.027	0.126**	-0.030	-0.071	0.054	0.044	0.034
Freshwater fish									1.000	0.279**	.099*	0.106*	0.152**	0.135**	0.142**	0.013	0.081	0.065	0.092*	0.093*
Marine fish										1.000	0.022	0.107*	0.090*	0.128**	0.065	-0.005	0.127**	0.112*	0.182**	0.109*
Egg											1.000	0.055	0.078	0.201**	-0.018	0.156**	0.122**	0.146**	0.059	0.150**
Leafy vegetables												1.000	0.184**	0.085	0.153**	0.090*	0.100*	-0.030	-0.036	0.004
Root and tuber crops													1.000	0.172**	-0.024	0.121**	0.174**	0.058	0.090*	0.126**
Fruit														1.000	0.027	0.108*	0.157**	0.135**	0.127**	0.060
Rice															1.000	-0.172**	-0.079	-0.069	-0.081	-0.017
Wheat																1.000	0.305**	0.004	-0.048	0.124**
Coarse grain																	1.000	0.140**	0.091*	0.145**
Milk or milk-products																		1.000	0.383**	0.161**
Yogurt																			1.000	0.151**
Soybean and its product																				1.000

*p<0.05, **p<0.01 significantly correlated with each other.

Table S3 Spearman's rank correlation between the FFQ and demographics

	Gender	Age	BMI	Drinking	Appetite for salty food	Physical exercise	Degree of satiety	Dietary pattern	Fast blood sugar(FBS)	Triglyceride
Vegetable oil	0.021	-0.036	-0.011	-0.005	-0.027	-0.109*	-0.008	0.046	-0.036	-0.008
Animal fats	-0.041	-0.200**	-0.009	0.006	0.047	0.026	-0.071	-0.113*	-0.012	0.003
Pork	-0.065	-0.159**	-0.008	-0.036	0.128**	-0.077	-0.099*	-0.268**	0.100*	0.017
Beef	-0.136**	-0.262**	0.005	0.052	0.090*	0.021	-0.050	-0.128**	-0.024	-0.032
Mutton	-0.116**	-0.072	0.082	0.100*	0.036	0.106*	-0.113*	-0.042	-0.023	-0.045
Poultry	-0.060	-0.239**	0.011	-0.031	0.040	-0.002	0.065	-0.102*	0.017	0.083
Gamey meat	-0.070	-0.072	-0.016	0.010	0.142**	0.013	-0.087*	-0.093*	-0.047	-0.033
Animal innards	-0.131**	-0.249**	-0.017	0.006	0.082	-0.070	0.006	-0.068	-0.008	-0.010
Freshwater fish	0.027	-0.271**	-0.014	-0.072	0.033	-0.012	-0.028	-0.032	0.043	-0.009
Marine fish	-0.061	-0.211**	-0.064	0.015	0.037	0.020	-0.043	-0.144**	0.012	0.051

Egg	-0.012	0.044	-0.089*	-0.049	-0.059	0.030	0.018	-0.002	0.004	-0.050
Leafy vegetables	0.050	0.005	-0.020	0.019	-0.094*	-0.035	0.009	0.119**	-0.012	0.076
Root and tuber crops	0.026	-0.126**	-0.041	-0.065	-0.058	0.043	-0.011	0.020	-0.049	-0.030
Fruit	0.094*	-0.060	-0.011	-0.077	-0.018	0.028	0.016	0.024	-0.023	-0.043
Rice	-0.076	-0.190**	-0.003	-0.050	0.093*	-0.135**	-0.082	-0.074	-0.044	0.055
Wheat	-0.094*	0.202**	0.117**	0.055	-0.034	0.088*	-0.007	-0.056	0.047	-0.035
Coarse grain	0.078	0.107*	0.062	0.012	-0.074	0.150**	0.095*	0.141**	0.063	-0.005
Milk or milk-products	-0.024	0.004	-0.046	-0.046	-0.007	0.065	-0.040	-0.013	-0.071	-0.126**
Yogurt	0.076	-0.076	-0.009	-0.051	0.005	0.103*	-0.001	0.030	-0.124**	-0.046
Soybean and its product	-0.029	-0.021	-0.047	0.009	0.047	0.026	-0.062	0.002	0.003	-0.080
Gender	1.000	-0.064	-0.174**	-0.509**	-0.101*	-0.033	0.078	0.141**	-0.002	0.102*
Age		1.000	0.121**	0.116**	0.030	0.158**	0.142**	0.067	0.085	0.068
BMI			1.000	0.155**	0.142**	-0.031	-0.050	-0.060	0.115**	0.084

Drinking	1.000	0.028	0.083	-0.033	-0.041	0.075	-0.099*
Appetite for salty food		1.000	-0.054	-0.081	-0.176**	-0.006	-0.060
Physical exercise			1.000	0.084	0.068	0.004	0.049
Degree of satiety				1.000	0.121**	0.020	0.096*
Dietary pattern					1.000	-0.049	0.010
Fast blood sugar(FBS)						1.000	0.023
Triglyceride							1.000

*p<0.05, **p<0.01 significantly correlated with each other.
